# Rural Reflections of Appalachia: A Qualitative Study of Health Professional Students’ Experiences from a Rural Immersion Experience in West Virginia

**DOI:** 10.13023/jah.0604.03

**Published:** 2025-01-29

**Authors:** Treah Haggerty, Jessica Stidham, Stephan Brooks, Abigail Cowher, Sandra Pope, Patricia Dekeseredy, Cara L. Sedney

**Affiliations:** West Virginia University Department of Family Medicine; West Virginia Institute for Community and Rural Health; West Virginia Institute for Community and Rural Health; West Virginia University

**Keywords:** Appalachia, immersion, medical education, medical students, rural communities, rural health, rural populations

## Abstract

**Introduction:**

Conventional academic rotations lack in-depth exposure to rural community members, systems, and resources surrounding specific rural-focused health issues.

**Purpose:**

This study aims to explore health professional students’ experiences within a community-based multidisciplinary rural immersion through their personal reflections.

**Methods:**

Written reflective entries were extracted from the online classroom system from 2019–2021. Data analysis was guided by thematic analysis. An iterative process of qualitatively coding the interviews was conducted. Themes were reviewed and agreed upon by consensus and assessed for validity by two senior researchers.

**Results:**

Sixty-two reflective essays were included from 11 unique professional programs. Resulting themes included (1) immersion was a transformative experience, (2) immersion experiences resulted in planned future practice changes, (3) immersion provided increased familiarity with stigmatizing diagnoses and contact with stigmatized groups, and (4) the experience provided knowledge of resources for future practice.

**Implications:**

Rural immersions provide a rich understanding of cultural identities, health systems, and health issues in a specific rural environment. Through the immersive experience, students identified future practice considerations, placed context to stigma and its effect on health, and concretely demonstrated interprofessional resources in rural clinical practice.

## INTRODUCTION

West Virginia (WV) is a largely rural Appalachian state known to have significant health disparities, such as high rates of heart disease, cancer mortality, and substance use disorder, leading to worse health outcomes when compared with its urban counterparts.^1^–^3^ This contributes to substantial overall morbidity and mortality.^1^,^4^ The heart disease mortality rate is 19% higher in WV than the national rate^5^, and the drug overdose death rate was 148% higher than the national average.^6^

Rural communities have unique cultural identities, health systems, and health needs. In spite of this, the vast majority of academic clinical rotations lack in-depth exposure to rural community members, systems, and resources surrounding specific rural-focused health issues. Medical students experience the health system primarily through their training environments, and even in rural states, most health professional training takes place in urban academic clinical settings^7^ due to the centralization of health professional student placements; because of this, they may not be aware of rural community-level issues impacting medical care and health. Common teaching methods to address this learning gap could include photo essays, various assignments and lectures, simulations, and case studies.^8^,^9^ A rural immersion experience, however, could better fill this gap.^10^,^11^

The first use of immersion for medical education occurred at the University of Minnesota Rural Physician Associate Program in 1971. To date, the program has 1600 alums, with 40% practicing in rural locations and 75% in primary care.^12^,^13^ Prior to its advent into medical education, the concept of immersion education originated in Canada in the 1960s, primarily as a way for students to learn a second language.^14^ In this teaching method, students are immersed in the social and cultural context; as such, the learning environment becomes the teaching tool. Immersion as an educational tool can include visual, auditory, kinesthetic, and simultaneous applications such as experiences in natural and cultural environments.^15^ Generally speaking, learning through an immersion experience helps students appreciate cultural differences and learn about the experiences of people from diverse backgrounds. Health professions education increasingly recognizes the value of integrating community-based, person-centered learning into the curriculum and how immersion techniques can benefit learners and community members.^10^,^11^ For example, rural immersions can engage health professional students or researchers in rural environments; this has the possibility of allowing an enhanced understanding of the rural community^16^ and improved engagement with that community. However, immersion needs to be done in a respectful manner as to avoid harm or fostering mistrust.^16^

The National Area Health Education Center (AHEC) program aims to enhance healthcare access by improving the supply and distribution of healthcare professionals in communities.^17^ The rural immersion module of WV’s AHEC is a component of the AHEC Scholars program within the national AHEC program. The primary purpose of the AHEC Scholars program is to “meet the needs of the communities they serve through robust community-academic partnerships, with a focus on exposure, education, and training of the current and future health care workforce.”^18^ This is accomplished through experiences that embed health professional students from various clinical training programs into rural communities to provide a community and health system-linked immersive experience. All health professional students are invited to participate. Upon completing this experience, students are asked to share their thoughts in a guided discussion and a written reflective questionnaire.

**Figure 1:**
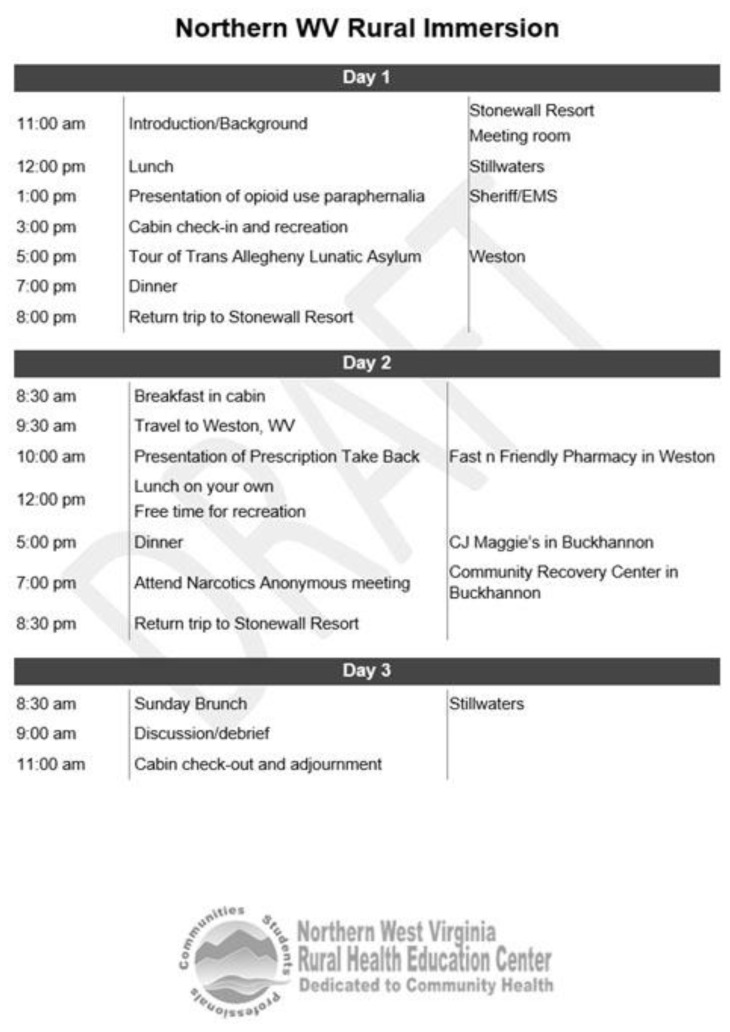
Example of a Rural Immersion Schedule

The AHEC Scholars program is an experiential learning program over two years that happens concurrently during selected student’s standard coursework. Rural immersion is a component of the AHEC Scholars program. Rural immersions include pre-work, such as reading pertinent academic articles and didactic education. In addition, a rural, on-location immersive experience is provided to the scholars across a period of two to three days. All interdisciplinary scholars taking part in the immersion travel to rural locations and are housed within the community during that time. There are five immersion options per year; students can choose to attend more than one, and locations can vary based on resources. Experiential learning focuses on cultural identity, health systems, and health needs of the community, and the following core topic areas are emphasized: (1) social determinants of health, (2) cultural competency, (3) interprofessional education, (4) behavioral health integration, (5) practice transformation, and (6) current and emerging health issues. Experts in the field represent local community partners and organizations, authors, health professionals, researchers, community service members (e.g., local law enforcement), community members, and AHEC Scholars faculty. Student participants are provided housing, food, and learning experiences at the location of interest. Experiences are planned purposefully to provide a broad understanding of a health issue and a detailed understanding of how the health issue affects a particular rural community or the rural area affects the health issue. A sample itinerary is shown in Figure 1.

The benefits of immersion education are well-studied; however, student perceptions of how this training can impact future health professional practice are less understood. This study aims to explore students’ experiences within a community-based multidisciplinary rural immersion to illuminate how this might affect their future practice. This is formative information to guide future training goals for improving the care provided by future health professionals to rural populations.

## METHODS

### Theoretical Framework

This immersion-based training provides a unique learning opportunity for health professional students consistent with Kolb’s learning theory. Kolb’s learning theory defines the most effective learner stages for information processing. The stages include 1) a concrete experience in the “DO” stage, 2) reflective observation in the “Watch and Listen” stage, 3) abstract conceptualization in the “Think” stage, and 4) active participation in the “Plan” stage.^19^ Kolb’s theory purports that learning occurs cyclically, encouraging continuous improvement. For our students, the immersion provided the concrete experience of being in a rural environment. This experience provides the context to reflect upon their observations and communications in order to conceptualize this learning and apply it to future behavior. This theory has proven useful for healthcare workers and has been utilized in a wide variety of interprofessional education programs^20^–^22^ and immersion experiences.^23^,^24^

### Participants

Health professions students from any West Virginia college or university who are currently enrolled in an eligible health professional program and have at least two years remaining in their degree program may participate in the AHEC Scholars Rural Immersion program. Eligible disciplines are listed in Table 1.

### Data Source

Students completed a reflective post-immersion discussion. Students answered these questions on a written discussion board in an online classroom environment. There was no open section for comments. Discussion prompts include the following five questions:

1. How did this immersion help you understand integrated primary care in the rural environment?
2. What did you learn from this immersion that was unexpected (a surprising fact, etc.)?
3. What is the most important aspect of the local environment affecting the population’s health?
4. Through your immersion experience, in what ways did you identify an interprofessional team addressing health?
5. What would be an innovative way to improve health through interprofessional teamwork in the environment in which you were immersed?

### Data Collection

Discussion board entries were extracted from the online classroom system from 2019–2021. Entries were anonymized by AHEC faculty before submitting to the study team for analysis.

### Data Analysis

The essays were uploaded to NVivo’s qualitative data analysis software to facilitate the analysis.^25^ Data analysis was guided by thematic analysis as outlined by Braun and Clarke.^26^ The transcripts were first read and reread by all investigators (T.H., A.C., P.D., C.S.), and initial ideas and thoughts were noted independently by each investigator. Initial codes were generated from this preliminary analysis. Following the initial coding, the investigators met to discuss their initial coding and to merge codes into overarching themes. This process was iterative until a consensus was reached on the candidate themes. Finally, the resultant themes were assessed by two senior researchers (TH and CS), reviewed for validity in relation to the transcripts, refined, and defined into four themes agreeable to all researchers.

### Ethics Approval

This project was approved by the West Virginia Institutional Review Board, protocol number 2204561210.

## RESULTS

Sixty-two reflective essays were reviewed. Fifty-five unique students were included in the analysis representing 11 professional programs. Some students took part in more than one immersion program. Professional programs and number of students per program can be found in Table 1. Four themes were identified through analysis of the student reflective essays: (1) immersion was a transformative experience; (2) immersion experiences resulted in planned future practice changes; (3) immersions provided increased familiarity with stigmatizing diagnoses and contact with stigmatized groups; and (4) immersions provided knowledge of resources for future practice.

### 1. Immersion was a transformative experience

The students described caring for the rural population and re-envisioning their future role in rural health care in a way not realized through their traditional curriculum. Prior to the experience, students completed an online didactic curriculum that taught them an overview of health care in the rural environment, social determinants of health and how they relate to the specific environment they would be in, and the economics of rural health care, which they reflected in their written responses.

“Prior to reading the numbers for health disparities in the Appalachian Region, I knew the numbers were grim, I have a better understanding of the why and the how to bring about change.” – Master of Social Work Student

The students credited the immersion with boosting their understanding of the far-reaching impact of rural health efforts. Students commented on the importance of policy in aiding rural health initiatives and the importance of patient-facing services on rural population health.

“Researchers and policy makers are working towards implementing programs to reduce these disparities and promote equality of health no matter a person’s geographic location, environment, or culture. This immersion made me realize how important it is for researchers and policy makers to implement these programs to improve the overall health of these rural communities.” – Bachelor of Science Nursing Student

Students perceived a greater understanding of how other health professions work collaboratively toward improved individual or population health goals. For example, they achieved a sense of how law enforcement approaches persons with opioid overdose.

“I was happy to hear that law enforcement often refers people who have been arrested for substance use or who have [overdosed] to hospitals and mental health services, rather than incarcerating them. I have learned the importance of various community members from different professions coming together to help solve the opioid epidemic and to better the lives of those affected by it.” – Doctor of Clinical Psychology Student

They also saw how health professionals from fields different from their own integrated into rural community health.

“Hearing from a pharmacist, a social worker, and a law enforcement personnel gave us insights that we would never have had if not for this immersion and I think this helps us develop strong interprofessional networking skills and recognize the value that other professionals bring to the table.” – Master of Public Health Student

Overall, students expressed a greater understanding in how different professions can work together to improve care in rural environments.

### 2. Immersion experiences resulted in planned future practice changes

Immersion experiences offer an opportunity to experience healthcare in a specific environment, which in turn shaped participant’s ideas of their own future practices.

“This will significantly alter the way I approach my work in the future, because it has taught me that just being a mental health provider is not enough. I also have a responsibility to advocate by reducing stigma through public psychoeducation as well as by lobbying to ensure that policymakers better understand the concerns the state faces and how their work impacts the people they serve.” – Doctor of Clinical Psychology Student

Students identified that the immersion gave them improved insight into the unique needs and characteristics of rural healthcare and how they will be cognizant of these needs when developing future practice, programs, and goals.

“In my future role as an epidemiologist, I am going to take everything I learnt in this immersion to become a holistic public health professional who has empathy, who values teamwork and takes into consideration every aspect of the society and rural health while designing interventions or conducting research.” – Master of Public Health Student

Students recognized the value of interprofessional work for the common goal of improving health and plan to incorporate interprofessional collaboration into their future practice caring for rural populations.

“After participating in these immersions, I am more likely to educate myself on local resources in my future area of practice and seek their assistance to meet patient needs.” - Doctor of Medicine Student

Students also endorsed a broadening of their way of communicating with patients in rural environments given their improved understanding of the broader context of rural health.

“This will alter the way I counsel patients or manage their treatment, is to focus on what extenuating circumstances may play a hidden role in the patient’s ailments, and to ensure that they comprehend and understand the nature of their disease process and to ensure that every opportunity or help they can receive to obtain their medications to optimize their treatment options.” - Doctor of Osteopathic Medicine Student

### 3. Immersion provided increased familiarity with stigmatizing diagnoses and contact with stigmatized groups

Students taking part in rural immersions described contact with stigmatized groups and improved familiarity of health issues at risk for stigma; frequently, this related to substance use disorder. The students experienced how stigma can affect individuals seeking healthcare.

“This experience has taught me that there is a lot of stigma around seeking treatment for opioid addiction.” – Doctor of Pharmacy Student

Through exposure to members of the rural community, students observed how stigma created worsening outcomes. Health outcomes are worse when individuals are not seeking healthcare secondary to stigma.

“She expressed there was a lot of stigma among first responders about seeking mental health treatment, though trauma and emotional fatigue were huge problems in the community, severe enough she had lost men to suicide.” – Doctor of Clinical Psychology Student

Understanding stigma and its effect on health can guide students’ plans to proactively address stigma in their future practice.

“Several people were uncomfortable with having a mental health screening, so it became clear to me that psychologists need to be doing more outreach and be involved in the community to help reduce the stigma related to seeking mental health services.” – Doctor of Clinical Psychology Student

### 4. Immersion experiences provided knowledge of resources for future practice

Students described how the lack of resources impacted residents of rural communities and how this was evident during their rural immersion experience. The indicated an increased awareness of how resources, both local and state, can be utilized to help fill gaps in healthcare needs.

“This experience taught me a lot more about resources outside of healthcare available to patients who suffer from substance abuse disorder which was incredibly helpful to me as a future provider.” - Physician Assistant Student

And this nursing student who felt a newfound appreciation for rural hardships.

“It will help me understand what type of resources my patients have that come from these types of areas and what kinds of things they could have gone through before they came to Morgantown. I think this will improve my care as a nurse and is an experience I will forever be grateful for.”- Nursing Student

Students also noted that interprofessional collaborations amplify the impact of local rural resources with the direct result of improving patient care. Through interactions with other health professionals within the immersion experience, students identified how other healthcare professionals provide essential services in rural areas.

“I also learned of additional resources which I need to consider as a part of my inter professional team. For example, I had never previously thought about social workers and how discussion with my patients, social worker can be helpful and important in understanding my patient.” – Doctor of Dentistry Surgery Student

Understanding of community resources and professionals within the health field is important for students future practice. These responses provide an example of how the immersion experience provided by the AHEC Scholars program illuminates the importance of immersive learning for both prospective providers and current educators.

## DISCUSSION

Rural environments are known to have increased health disparities compared to their urban counterparts; however, health professional students’ exposure to these disparities is comparatively less while in training at academic medical centers. Rural immersion-based training helps students identify future professional^10^ and practice transformation to implement for improved patient care outcomes. Our students shared how being in the community, talking with residents, and seeing how they live shaped their thoughts about rural practice. Moreover, these experiences enhance students’ appreciation of how interprofessional collaborations are both feasible and effective in rural environments. Students in this study reported how participating in immersion-based training was important preparation for future rural practice.

Rural immersion experiences, such as the one offered through AHEC, have been successfully implemented into training within various fields. Using principles from Mennenga, et al.’s nursing rural telehealth simulation^27^ and Kolb’s experiential learning theory^28^, the University of Queensland (Australia) implemented a rural simulation activity for their second-year physiotherapy students. The three-part experience included an orientation to the patient’s home environment, their commute to a healthcare facility, and an encounter with a rural standardized patient. A structured debriefing where students were asked to consider multiple factors including rural environments, rural health risks, rural healthcare access, technology, rural practice components, and rural culture was included. The activity achieved its aim of increasing intrapersonal empathy toward rural populations. Additionally, in group discussions, it was found that students became more aware of the challenges and adaptations associated with rural healthcare through the simulation and that they were satisfied with the experience.^29^

Through immersion experiences, students are challenged to change previously held beliefs about unfamiliar practice environments. This transformative change can create a newfound focus on rural health, as seen in some of our students, or a more focused or realistic view of rural health practice amongst students already committed to rural health care. This has also been seen in other immersion experiences. To inspire more dental school graduates to practice in rural areas, the University of Minnesota School of Dentistry began offering a three-week mentorship with a dentist in rural practice to immerse students in the realities of rural dentistry. They utilized transcripts of focus groups, student reflections, and personal statements from students’ dental school applications to evaluate the program’s success. They found that the program succeeded in correcting a myriad of misconceptions, highlighting positive features of rural practice and emphasizing the importance of dentists in rural areas. Some reformed misconceptions were that rural communities lacked opportunities for professional development, were unwelcoming toward newcomers, and had limited diversity. A few benefits of rural practice emphasized by students were the meaningful interpersonal relationships and rural dentistry’s busy, profitable nature. In addition, regarding the essentiality of dentists in rural areas, one student noted that her mentor came out of retirement because the need for a dentist in the area was so dire, while another stated the frustration associated with a three-month-long waiting list for an appointment.^30^

In addition to interacting with the health professionals in the rural community, our students had the opportunity to interact with community members. This experience often led to a “contact intervention” amongst potentially stigmatized groups. Stigma has been documented across multiple health conditions, including substance use disorder, HIV, and mental health conditions, and has a negative impact on health-related domains. Stigma towards disorders commonly represented in rural environments, such as that related to substance use disorder, may be proactively addressed through contact interventions with the stigmatized groups. Our participants emphasized the impact of such contact exposure with stigmatized groups during their immersion experiences. Immersion experiences are well known within medical education and include both a contact and an educational component, mirroring the two primary stigma mitigation interventions for which research is available. Several previous studies have assessed the mitigation of stigma within professional groups and found that both contact and educational interventions may be effective.^31^–^33^

Immersive experiences also drive the transformation of future practice as suggested by our participants. Other similar programs have also described this. In South Carolina, a Rural Interdisciplinary Practicum was designed for students in nursing, health administration, medicine, occupational therapy, pharmacy, and physical therapy, in which they lived and worked in a rural area for five weeks. Program components included reading assignments, didactic components, immersion-based training through pairing with a preceptor in clinical practice, field trips to community events, and interdisciplinary case studies. The program produced a variety of positive outcomes. Students became more aware of the barriers to healthcare in rural areas by witnessing increased transportation time to facilities due to distance and two-lane highways shared with log trucks and tractors. They likewise became more aware of the perspectives of other disciplines and gained other valuable knowledge. The majority of participants were also positively influenced in their intention to pursue rural-based practice.^34^

This qualitative analysis does have some limitations. The present experience placed students in rural West Virginia, which could limit the generalizability of findings to other rural placements. This was an analysis of written work completed for a class assignment. The students may have been biased to report positive outcomes. The discussion prompt questions could also be read to elicit positive responses that lead to thematic outcomes. Deeper insight would be gained from in-depth interviews of participants focusing on the prevalent themes. Additionally, there is presently no way of verifying with the current methodology that future planned practice changes occurred or that the impact of the experience persisted in the long term; this information is somewhat limited by the length of time students spend in the community. Longer immersion experiences could produce more robust results. Long-term follow-up of students could provide insight into lasting changes.

## IMPLICATIONS

Rural immersions for healthcare students can provide a rich understanding of cultural identities, health systems, and health issues prevalent to specific areas, such as Appalachia. Moreover, it presents the student participants with insight into the benefits of rural practice. Through immersive experiences, students identify future practice considerations, provide context to stigma and its effect on health, and concretely demonstrate interprofessional resources in rural clinical practice. This education is especially useful in healthcare and may encourage graduates to practice in areas with health professional shortages.

**SUMMARY BOX**

**What is already known about this topic?**

Most health professional training takes place in urban academic clinical settings. As such, conventional academic clinical rotations lack in-depth exposure to rural community members, systems, and resources surrounding specific rural-focused health issues.

**What is added by this report?**

The benefits of immersion education are well studied; however, student perceptions of such training on future health professional practice are less understood. This study explores students’ perceptions of a community-based multidisciplinary rural immersion to know how this experience might impact their future practice.

**What are the implications for future research?**

Rural immersions for healthcare students can provide a rich understanding of cultural identities, health systems, and health issues prevalent in specific regions, such as Appalachia. These results are beneficial in informing curriculum development for healthcare educators and may encourage graduates to practice in medically underserved areas. Future research should include long-term follow-up with former participants to provide insight into lasting change.

## Figures and Tables

**Table 1:** Health Professions Included in the WV AHEC Rural Scholars Program from 2019–2021 and Analysis

Professional Program	Population N (%)
Dentistry (DDS)	4 (7.27)
Dental Hygiene (AS, BS)	0
Exercise Physiology (BS)	4 (7.27)
Health Informatics and Information Management (BS, MS)	0
Medicine (MD, DO)	12 (21.82)
Public Health (BS, MPH)	3 (5.45)
Nursing (AND/ASN, BSN, DNP, PhD)	7 (12.73)
Nutrition/Dietetics (BS, MS)	3 (5.45)
Occupational Therapy (MOT, DOT)	1 (2.82)
Pharmacy (Pharm.D, PhD)	9 (16.36)
Physical Therapy (DPT)	0
Physician Assistant (MPA)	3 (5.45)
Psychology (MA, PhD, Psy.D)	8 (14.55)
Social Work (BSW, MSW)	1 (2.82)
Speech-Language Pathology (MSPA)	0
